# Dietary glycaemic index and glycaemic load among Australian adults – results from the 2011–2012 Australian Health Survey

**DOI:** 10.1038/srep43882

**Published:** 2017-03-06

**Authors:** Jimmy Chun Yu Louie, Molly Jones, Alan W. Barclay, Jennie C. Brand-Miller

**Affiliations:** 1 School of Life and Environmental Sciences, Faculty of Science, The University of Sydney NSW 2006, Australia; 2 Charles Perkins Centre, The University of Sydney NSW 2006, Australia; 3 School of Biological Sciences, Faculty of Science, The University of Hong Kong, Pokfulam, Hong Kong; 4 Accredited Practising Dietitian, Sydney, Australia

## Abstract

This study aimed to determine the major food groups contributing to dietary glycaemic load (GL). Plausible food intake data collected using a multiple-pass 24 hour recall from a weighted sample of 6326 adult respondents (52% male) of the 2011–2012 Australian Health Survey dataset (AHS) were analysed. The GI of foods was estimated based on a previously published step-wise method. Descriptive statistics were calculated for dietary glycaemic index (GI), GL and contribution to GL by major food groups, stratified by age and sex. Trends across age groups were assessed using linear regression. Pearson’s χ^2^ was used to test for differences between age groups for categorical demographics variables. The mean (SD) dietary GI and GL was 54 (7) and 135 (59) respectively and the top 3 contributors to dietary GL were breads (14.4%), cereal-based dishes (10.3%) and breakfast cereals (ready to eat) (6.6%). There were small but significant differences in the GL contribution pattern between the sexes. The findings indicate that the average dietary GI of Australian adults is similar to that of other population groups, with a large proportion of starchy and energy-dense nutrient-poor foods that contribute to a high GL.

Evidence from recent meta-analyses^1^,^2^,^3^,^4^,^5^,^6^,^7^ suggests that a low glycaemic index (GI) and/or glycaemic load (GL) eating pattern is associated with better health outcomes, such as decreased risk of type 2 diabetes, coronary heart disease, gallbladder disease, and breast cancer. This is not true for carbohydrate content alone. Glycaemic load, the product of carbohydrate content and the GI, has been shown to be the best predictor of postprandial glycaemia and insulinaemia^8^, two factors that are intrinsically involved in the pathogenesis of chronic disease^9^. Dietary interventions to lower the dietary GI and/or GL may therefore be effective in promoting population health.

To better design appropriate dietary intervention strategies to lower the dietary GI and GL, it is important to know the current levels and contributors to dietary GI/GL of the target population. Until recently, nationally representative data for Australian adults were scarce. Several Australian population-based cohort studies^10^,^11^,^12^ have reported dietary GI and GL of their respective sub-populations. For example, Barclay *et al*.^10^ reported that the average dietary GI and GL among a group of 2,736 older Australian adults aged 49 years or above was 57 (SD 4) and 134 (SD 24) respectively, while O’Sullivan *et al*.^11^ reported a similar average dietary GI of 56 (SD 4) but a lower average dietary GL of 115 (SD 25) in a group of 459 women aged 42–81 years. While these studies provide useful insights into the current levels and contributors to dietary GI/GL, the findings are not generalisable to the entire Australian population. They cannot be used to inform policy development as there are likely to be variations in dietary GI and/or GL, and their contributors, according to age, sex and socioeconomic status. For example, it is likely that people with low socioeconomic status consume cheaper foods^13^ such as white bread which has a high GI.

Australia has conducted several national nutrition surveys in the past 40 years^14^,^15^, but no previous attempt has been made to utilise these data to examine the dietary GI and GL (and their contributors) of the general Australian adult population. Our group has previously examined this in the Australian children and adolescents population using data from the 2007 Australian National Children’s Nutrition and Physical Activity Survey^16^. We found there were key foods such as breads and cereal-based dishes contributing to a high GI/GL eating pattern amongst Australian children and adolescents. Whether the pattern is similar in Australian adults, or not, will inform the development of health promotion strategies and policies. As GL is a function of both carbohydrate quality, i.e. GI, and quantity, it is helpful to examine the age and sex variations in GL since younger adults tend to have higher overall energy and hence carbohydrate intake than their older counterparts, and the same is true for men *vs*. women of the same age.

Therefore, using data from the recently released Australian Health Survey (AHS)^14^, we aimed to describe the current dietary GI and GL, and explore the main food sources contributing to the dietary GL of a nationally representative sample of Australian adults according to age and sex.

## Results

The characteristics of the participants are summarized in Table 1. The mean ± SD BMI of the study population was 26.8 ± 5.8 kg/m^2^, with 37.6% and 22.5% considered overweight and obese, respectively. Extreme low-reporters excluded from the analysis were similar mean age to those included (47 ± 17 *vs*. 46 ± 18 years; *p* = 0.589) and had a higher mean BMI of 29.7 ± 6.3 kg/m^2^ (*p* < 0.001); whereas extreme high-reporters excluded had a significantly lower age of 40 ± 17 years (mean ± SD, *p* < 0.001), and lower BMI of 23.1 ± 4.3 kg/m^2^ (*p* < 0.001), compared with participants with plausible dietary intake. Respondents excluded due to missing weight data were significantly older (49 ± 18 years; *p* = 0.007).

The dietary intake of the subjects, stratified by age groups, is summarized in Table 2. The mean ± SD dietary GI and GL of adult participants were 53.9 ± 6.8 and 135.4 ± 58.5 respectively. There were decreasing linear trends of dietary GI and GL as age increases (both *p* < 0.001).

Bread and bread rolls (14.4%); cereal-based dishes (10.3%); and breakfast cereals (ready to eat) (6.8%) were identified as the top 3 contributors to dietary GL, accounting for nearly 40% of the dietary GL on a *per capita* basis. The ranking of GL contributions per food group varied considerably among age groups (Supplementary Table 6).

Table 3 summarises the *per capita* contribution to dietary GL by the top 20 contributors, stratified by age group. Cereal-based dishes; flours, cereals and starches; sweetened beverages; fruit and vegetable juices and drinks; poultry-based dishes; and pastas all contributed less to dietary GL as age increased, while bread and bread rolls; breakfast cereals (ready to eat); sugar, honey and syrup; cake-type desserts; sweet biscuits; dairy milk; tropical and subtropical fruit; pome fruit; savoury biscuits; and frozen milk products contributed more to dietary GL as age increased (all *p*_trend_ < 0.001). *Post hoc* comparisons (all *p* < 0.001) indicated that adults aged 19–30 years had significantly lower proportions of dietary GL from bread and bread rolls, and sugars, honey and syrups; and significantly higher proportions of dietary GL from cereal-based dishes, sweetened beverages and fruits and vegetables juices and cordials, compared with their older counterparts. They also had significantly higher proportions of dietary GL from poultry-based dishes and pasta compared with adults aged 51 years or above, and a significantly lower proportion of dietary GL from dairy milk.

When examined by sex (Supplementary Table 7), significant differences in percentage GL contribution between sexes (all *p* < 0.001) were observed for breakfast cereals (ready to eat) (M: 7.1 ± 11.4% *vs*. F: 6.1 ± 10.5%); flours, cereals and starches (M: 7.0 ± 16.5% *vs*. F: 5.6 ± 14.4%); sweetened beverages (M: 5.1 ± 10.3% *vs*. F: 3.2 ± 8.9%); cake-type desserts (M: 3.1 ± 8.5% *vs*. F: 4.7 ± 11.2%); tropical and subtropical fruit (M: 1.8 ± 4.5% *vs*. F: 2.5 ± 5.8%); pome fruit (M: 1.6 ± 3.8% *vs*. F: 2.0 ± 4.2%); savoury biscuits (M: 1.4 ± 5.2% *vs*. F: 2.1 ± 6.4%); and chocolates (M: 1.3 ± 4.7% *vs*. F: 1.9 ± 5.9%). Percentage GL contributed from fancy breads was also marginally different between sexes (M: 2.1 ± 7.0% *vs*. F: 2.7 ± 7.8%; *p* = 0.001).

*Per consumer* analyses, as presented in Table 4, revealed a different picture. Positive trends with age were observed for breads and bread rolls, and sugar, honey and syrups (both *p*_trend_ < 0.001); while decreasing trends with age were observed for breakfast cereals (ready to eat); flours, cereals and starches; sweetened beverages; and fruit and vegetable juices and drinks (all *p*_trend_ < 0.001). Interestingly, *post hoc* analysis revealed that these trends across age groups were mostly driven by the significant difference between the 19–30 year olds and their older counterparts.

When examined by sex (Supplementary Table 8), significant differences between sexes (all *p* < 0.001) were observed for flours, cereals and starches (M: 35.5 ± 19.3% *vs*. F: 30.5 ± 19.1%); sweetened beverages (M: 15.0 ± 12.9% *vs*. F: 12.9 ± 13.8%); cake-type desserts (M: 19.1 ± 11.6% *vs*. F: 23.0 ± 14.3%); tropical and subtropical fruit (M: 9.0 ± 6.0% *vs*. F: 10.5 ± 7.3%); and pome fruit (M: 7.0 ± 4.9% *vs*. F: 7.9 ± 4.8%). Percentage GL contributed from chocolates was marginally different between sexes (M: 7.7 ± 9.1% *vs*. F: 9.6 ± 10.2%; *p* = 0.003).

Energy- and SEIFA adjustments to the analyses did not materially alter the interpretations and conclusions (Supplementary Tables 9 and 10).

## Discussion

This study is the first to report the GI and GL of a nationally representative sample of Australian adults. Overall, the mean GI and GL of participants was 54 (SD 7) and 135 (SD 59), respectively. Both GI and GL decreased with advancing age. Common carbohydrate-rich staple foods including bread and bread rolls, cereal-based dishes and breakfast cereals (ready to eat) were the top contributors to dietary GL in Australian adults.

Our group had previously reported data on children and adolescent respondents from the same national survey^17^. Comparing the results of adults versus children and adolescents using one-way ANOVA, the latter had significantly higher dietary GI (55.5 ± 5.3 *vs*. 53.9 ± 6.8; *p* < 0.001) and dietary GL per MJ (16.7 ± 3.3 *vs*. 14.1 ± 4.2; *p* < 0.001), but lower fibre density (2.5 ± 1.0 g *vs*. 2.7 ± 1.2 g; *p* < 0.001). We also observed significant differences (one-way ANOVA, all *p* < 0.001) in the per consumer percentage GL contribution from different food groups between adults and children and adolescents. Adults had higher proportions of their dietary GL contributed by flours, cereals and starches (6.3% *vs*. 4.6%); potatoes (5.4% *vs*. 4.5%); sugars, honey and syrups (4.0% *vs*. 1.7%); and pastries (2.5% *vs*. 1.8%); and lower proportion of their dietary GL from fruit and vegetable juices and drinks (2.8% *vs*. 3.9%); sweet biscuits (2.2% *vs*. 3.2%); dairy milk (2.1% *vs*. 2.9%); pome fruit (1.8% *vs*. 2.6%); savoury biscuits (1.7% *vs*. 3.0%); and frozen milk products (1.1% *vs*. 1.5%). Furthermore, in the current study, older adults had a large proportion of their dietary GL in the form of multigrain bread and bread rolls which have a lower GI than conventional breads, as also reported by Barclay *et al*.^10^. Flours, cereals and starches, and cereal-based dishes contributed significantly less to the diet of older adults. However, among other age groups, there were substantial differences in the proportion of GL contributed by different food groups. A higher proportion came from low or moderate GI foods such as biscuits, cakes, sugars and fruit. We also observed small but significant differences between the sexes. These findings suggest that a one-size-fits-all strategy to lower the populations dietary GI and GL is unlikely to be effective.

When comparing dietary GI and GL data from different international studies, it is important to take the dietary assessment methods used into account. For example, the study by Murakami *et al*.^18^ in British adults utilized a 7-day weighed food record as the choice of dietary assessment, which is regarded as the gold standard^19^. While the reported dietary GL of 140 is comparable to our results, the dietary GI was higher at 59. A study by Lin *et al*.^20^ which utilized dietary data collected from two 24 hr recalls found the mean dietary GI and GL of the American population to be 56 and 138, slightly higher than what we observed in the Australian population. On the other hand, other population studies^21^,^22^ using semi-quantitative FFQs reported lower dietary GLs despite similar dietary GI. Whether these differences are due to true differences between the sample populations, or the choice of dietary assessment methods, or both, is difficult to ascertain.

Our results also imply that older adults (aged 51 years or above) in the Australian population have a similar dietary GI (53 *vs*. 54) but lower GL (123 *vs*. 135) than that assessed by a semi-quantitative FFQ in a sub-population of 2,900 Australian adults in the Blue Mountains region^10^. In contrast, we report a higher dietary GL (135 *vs*. 115) and lower dietary GI (54 *vs*. 56) than that assessed using a diet history in a small sample of 459 women in Brisbane Australia. Again, differences in the dietary assessment methods and the different periods of data collection may have contributed to these disparate findings. Clearly, nationally representative data much better inform policy discussion.

On a *per capita* basis, breads and bread rolls were the highest contributor to GL (14.4%), followed by cereal-based dishes (10.3%), breakfast cereals (ready to eat) (6.6%), flours, cereals and grains (6.3%), potatoes (5.4%) and sweetened beverages (4.2%). To inform public health strategies and policy discussions regarding lowering dietary GI and GL, it is important to look at both the *per consumer* and *per capita* data, where the *per capita* data provide a snapshot of the population, and the *per consumer* data provide the actual intake level and pattern for those who consume a higher GI/GL diet. The *per consumer* analysis revealed that flours, cereal and starches were higher contributors (33.2%), followed by cereal-based dishes (29.1%), breads (21.9%), cake-type desserts (21.1%), potatoes (16.3%) and pasta (21.6%). The large differences between the *per capita vs. per consumer* results suggests that the *per capita* data were likely skewed to the lower end by a large number of non-consumers. Hence to better target individual-based dietary intervention strategies, the *per consumer* data should be used. *Per capita* data should be used for developing population-based strategies.

It is often assumed that a low GI diet is one with a GI less than 55 given low GI foods are defined as those that have a GI of 55 or less^23^. However, epidemiological research suggests that a dietary GI of 45 or below is associated with reduced risk of chronic diseases such as diabetes^23^,^24^,^25^. Our results indicate that only 9% of Australian adults are actually meeting this target. According to the definition, dietary GI is a weighted average GI of all carbohydrate foods in the diet. Hence to effectively lower dietary GI, individuals should be advised to select low/lower GI staple foods as their main sources of carbohydrate. Common examples include dense wholegrain breads (GI ~55) over white bread (GI ~70); traditional rolled oats (GI ~50) over cornflakes (GI ~80) or puffed rice (GI ~85); and basmati rice (GI ~55) over jasmine rice (GI ~80). Replacing some nutrient-dilute starchy foods and snacks such as white rice or rice crackers in the diet with fruits and/or reduced fat dairy products (which are mostly low GI) will help lower GI as well as improve micronutrient intake.

While dietary GI represents the quality of carbohydrates in the diet, dietary GL which takes into account both quantity and quality of the dietary carbohydrates was found to better predict postprandial glycaemia and insulinaemia^8^. Livesey *et al*.^26^ previously reported that a dietary GL below 95 per 2000 kcal (i.e. ~114 per 10 MJ) was associated with a significant reduction in risk of type 2 diabetes. We found that only 0.5% of the study population were able to meet this cut-off. Since the carbohydrate intake of Australians was not high, this suggests that Australians should be selecting more low GI foods to lower the dietary GL to reduce their risk of chronic diseases.

The use of a published method^27^,^28^ to assign GI values to food is a major strength of the current study, as the process of assigning GI is reproducible and transparent. The use of this standard approach enables between-studies comparison. A sensitivity analysis was also conducted which ensures our results were not biased due to exclusion of extreme under- and over-reporters. The use of a nationally representative sample also enables our findings to be applied in other settings in Australia.

Our study is however limited in several ways. First, we used only the first 24 hour recall, which is unlikely to capture the usual dietary pattern of an individual^29^, although the use of one 24 hour recall is commonly used and deemed appropriate for estimating population means^30^. Using only the first 24 hour recall collected in a face-to-face interview also reduced the impact of under-reporting associated with telephone interviews^31^, which was the method used in the second 24 hour recall. We however found large SDs for the mean dietary GL and %GL contribution, similar to our previous analysis in children and adolescents in the same national survey^17^. While this suggests wide inter-individual variation, meaning a substantial proportion of the population were consuming a high GL on the day of survey despite a moderate population mean, it could also be a result of the intra-individual day-to-day variability in dietary intake which is widely acknowledged^30^. Using multiple 24 hour recalls or statistical approaches to account for such variability would produce better estimate of the true variability in dietary GL in the population. Nonetheless, the inherent limitation of the 24 hour recall method, i.e. heavy reliance on the respondent’s ability to correctly recall the foods and beverages consumed in the past 24 hours, which may lead to memory bias, should not be overlooked. Second, we have grouped foods based on the pre-defined minor food classification^32^ which was not developed according to the GI of foods. This meant we were unable to differentiate similar foods with different GI, e.g. wholemeal breads (high GI) *vs*. dense multi-grain breads (low GI), and as such we were unable to tell whether the high contribution of breads to dietary GL is due to the choice of high GI breads, of the high consumption of breads in general, or both. However, this limitation likely only affected food groups with considerable within group variability such as breads and cereals. Using the pre-defined food classification allows better comparisons of other secondary analyses of the Australian Health Survey, and the results will be more interpretable by dietitians and public health practitioners who are familiar with the survey.

In conclusion, the average dietary GI of Australian adults was found to be substantially higher than that associated with reduced risk of chronic disease in other populations. Significant age and sex differences in GL contribution pattern were also observed. Breads remain the top contributor to dietary GL among Australian adults, while younger adults are deriving more of their dietary GL from a wide range of foods, especially processed foods. Dietary interventions aiming at reducing dietary GI and/or GL should hence be age- and sex-specific, where interventions aiming at elderly should focus on swapping high GI staple food with low GI versions, while for younger adults discouraging consumption of high GI processed foods may be more effective.

## Methods

### Data source

This analysis used data from the National Nutrition and Physical Activity Survey (NNPAS) component of the 2011–2012 AHS. The AHS was conducted by the Australian Bureau of Statistics (ABS) between 2011 and 2012, with a response rate of 77%^14^. The methodology of the NNPAS was previously described in detail^33^. The survey collected information on the dietary intakes of food and beverages, as well as the use of supplements, using the computer-assisted, multiple-pass 24 hour recall method.

### Anthropometric measurements

Weight and height were measured without shoes and heavy clothing where possible, using a digital scale and a stadiometer, to the nearest 0.1 kg and 0.1 cm, respectively. Body mass index (BMI) was calculated as weight in kg divided by the square of height in meters. Participants were then classified into underweight, normal weight, overweight or obese based on the following BMI cut-offs: underweight ( ≤ 18.5 kg/m^2^); normal weight (18.5–24.99 kg/m^2^), overweight (25.0–29.99 kg/m^2^) and obese (≥30.0 kg/m^2^)^34^.

### Dietary assessment

All participants aged 19 to 95 years (*n* = 9,313) had dietary information collected in a face-to-face interview between 29 May 2011 and 9 June 2012. A phone interview was conducted in ~60% of the participants at least 8 days after the face-to-face interview to collect a second 24 h recall. A two dimensional food model booklet was provided to the respondent to aid portion size estimation^35^. Dietary intake data were entered into a purpose-built database, and were translated into nutrient intake using the AUSNUT2011-2013 database^36^. A method previously described by our group was used to assign GI values to individual food items in AUSNUT2011-2013^27^,^28^. In brief, foods with ≤2.5 g available carbohydrates per 100 g were assigned a GI value of 0 (Step 1, *n* = 1752). All remaining foods had their GI values assigned based on a step-wise, systematic approach, using either the GI value of an exact match (Step 2, *n* = 363) or a closest match (Step 3, *n* = 1738) in one of the 4 commonly used GI databases^37^,^38^,^39^,^40^. Foods without a match in these tables were either assigned a weighted GI value if they were mixed dishes which could be disassembled into individual ingredients (Step 4, *n* = 1526), or the median GI of the corresponding food subgroup (Step 5, *n* = 205). A small proportion (*n* = 60) of foods were assigned a default GI as none of the earlier steps could be used to assign a GI to them.

Foods were grouped together based on the minor food groups in AUSNUT2011-2013^32^ to enable identification of the main food sources contributing to dietary GL. The AUSNUT food groups were renamed and shortened for better presentation in text and tables (Supplementary Table 1)^17^.

### Calculation of dietary glycaemic index and glycaemic load

The GL of each food was calculated by GI (%) × available carbohydrates (in grams) in a serving of that specific food. The daily dietary GL of each subject was then calculated as the sum of GL from all foods consumed in that day. Dietary GI was calculated by the following formula: (dietary GL/total available CHO intake in the day) × 100^16^,^41^.

### Data cleaning

In this study, only the first 24 h recall collected from a face-to-face interview were used. This is likely to reduce the influence of under-reporting which is common in the follow-up recall via phone interview^42^. The plausibility of the food intake data was assessed using the equation proposed by Goldberg *et al*.^43^ to exclude extreme low- and high-reporters. As the survey did not provide data on physical activity level (PAL), the standard PAL of 1.55 was used in the calculation of the cut-offs, as per the AHS recommendations^33^. The lower 95% confidence interval (CI) of PAL of 1.55 (i.e. 0.87) and the upper 95% CI (i.e. 2.76) were used as the cut-off values for extreme low- and high- reporters, respectively^43^. Based on this method, 1593 extreme low reporters and 124 extreme high reporters were excluded. We also excluded 1318 respondents as they did not have their weight recorded which disallowed the computation of the energy intake to basal metabolic rate (EI:BMR) ratio. A sensitivity analysis was performed where results of all participants and only those with plausible data were compared, and no material differences in the direction of trends and conclusions were observed (Supplementary Tables 2–4). Hence the extreme mis-reporters were excluded in the final analysis. The final weighted dataset included 6326 individuals (unweighted *n* = 6278), of which 52.4% were male.

### Statistical analysis

The AHS had over-sampled males to females, and had a much larger percentage of adults than children. Furthermore, major city residents were over-represented^14^. Sample weighting was applied to the dataset to allow generalization of the findings to the general Australian population^33^,^44^. Data were presented as mean (SD) for continuous variables, and as percentages for categorical variables. The number of consumers for each age group and sex for the top 20 food groups contributing to GL was counted (see Supplementary Table 3). The percentage that each food group contributed to the dietary GL was calculated as 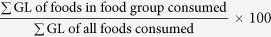. For the food sources analyses, *per capita* and *per consumer* results were both presented. *Per capita* analyses included all participants and provide a snapshot of an average individual, whilst *per consumer* analyses included only the participants who had reported consumption of the specific food group on the day of the food recall. The latter provided information on average serving size. Age groups pre-defined in the AHS were used in the stratified analysis to allow better comparisons across surveys within Australia, and *post*-*hoc* comparisons between age groups were performed by using the Bonferroni *post*-*hoc* test for one-way ANOVA. Differences between sexes for each age group were tested by one-way ANOVA. Linear regression was used to test for trends across age groups. All main statistical analyses were carried out using SPSS version 22.0 (IBM, California, USA). Additional analyses adjusted for energy and SEIFA were performed using General Linear Model in SPSS Complex Samples. A *p* value of <0.001 was considered to be statistically significant to minimize type I error^45^.

## Additional Information

**How to cite this article:** Louie, J. C. Y. *et al*. Dietary glycaemic index and glycaemic load among Australian adults – results from the 2011–2012 Australian Health Survey. *Sci. Rep.* **7**, 43882; doi: 10.1038/srep43882 (2017).

**Publisher's note:** Springer Nature remains neutral with regard to jurisdictional claims in published maps and institutional affiliations.

## Supplementary Material

Supplementary Tables

## Figures and Tables

**Table 1:** Participant characteristics, stratified by age group.

	19–30 years	31–50 years	51–70 years	71 + years	^1^*p* value
*n*	1523	2345	1792	666	—
BMI (kg/m^2^)^2^	24.8 ± 4.8	26.9 ± 5.0	28.2 ± 5.1	27.2 ± 4.7	<0.001
*Under weight* (*%*)	3.5	1.1	1.2	1.5	<0.001
*Normal weight (%*)	55.8	37.7	26.9	30.0	
*Overweight (%*)	29.0	38.8	40.5	45.1	
*Obese (%*)	11.7	22.4	31.4	23.3	
Male (%)	56.0	52.7	51.3	46.4	0.006
Living in urban area (%)	79.1	72.8	63.7	69.7	<0.001
Proportion in the highest SEIFA quintile (%)	23.3	25.3	22.5	19.2	0.013
Proportion in the highest decile of household income (%)^3^	10.6	14.8	12.7	2.8	<0.001
Born in Australia (%)	70.5	68.0	68.3	71.8	0.135

Values were presented as mean ± SD for continuous variables and as percentages for categorical variables. Data were weighted to account for over- or under-sampling to enable representation of the general Australian population, and the *n* reported in the table represent the weighted sample. SEIFA - Socio-Economic Indexes for Areas, a measure of socioeconomic status defined by the Australian Bureau of Statistics, where a higher SEIFA indicate socio-economic advantage^46^.

^1^*p* values represent *p* for trend across age groups tested by linear regression for continuous variables, and for categorical variables the *p* values were tested by χ^2^ test.

^2^*n* = 1522, 2340, 1787, 649 respectively due to missing data.

^3^*n* = 1184, 2227, 1649, 598 respectively due to missing data.

**Table 2:** Dietary intake of participants, stratified by age group.

	19–30 years	31–50 years	51–70 years	71 + years	^*1*^*p*_trend_
Dietary GI	54.9 ± 6.6	54.0 ± 6.7	53.2 ± 7.2	53.2 ± 6.0	<0.001
Dietary GL	154.5 ± 62.8	137.5 ± 56.5	124.6 ± 56.7	113.4 ± 44.3	<0.001
Dietary GL/MJ	15.0 ± 4.2	14.1 ± 4.2	13.4 ± 4.3	14.0 ± 3.7	<0.001
Dietary GI < 45 (%)	9.1	10.1	12.6	11.0	0.009
Energy (kJ)	10342 ± 3063	9839 ± 3095	9351 ± 2851	8086 ± 2326	<0.001
Energy from fat (%)	31.8 ± 9.1	31.4 ± 8.3	31.2 ± 8.7	30.7 ± 8.3	<0.001
Energy from saturated fat (%)	12.6 ± 4.7	12.4 ± 4.5	12.1 ± 4.8	12.6 ± 5.0	0.061
Energy from protein (%)	17.4 ± 5.7	18.0 ± 5.6	18.0 ± 5.5	17.9 ± 5.1	0.025
Energy from carbohydrates (%)	45.0 ± 10.0	43.0 ± 10.4	41.3 ± 10.8	43.6 ± 9.7	<0.001
Energy from total sugars (%)	19.5 ± 8.5	18.9 ± 8.8	18.2 ± 8.4	20.3 ± 8.0	0.491
Energy from starch (%)	24.7 ± 9.5	23.2 ± 9.3	22.1 ± 9.2	22.4 ± 7.6	<0.001
Fibre density (g/MJ)	2.4 ± 1.1	2.6 ± 1.1	2.8 ± 1.2	3.2 ± 1.3	<0.001

Values were presented as mean ± SD for continuous variables and as percentages for categorical variables. Data were weighted to account for over- or under-sampling to enable representation of the general Australian population.

^1^*p* values represent *p* for trend across age groups tested by linear regression for continuous variables, and for categorical variables the *p* values were tested by *χ*^2^ test.

**Table 3:** Per-capita percentage dietary glycaemic load contribution from the top 20 food groups, stratified by age group.

Food groups	19–30 years	31–50 years	51–70 years	71 + years	*p*_trend_^1^
Bread and bread rolls	10.8 ± 13.2	13.6 ± 15.0^†^	16.6 ± 16.4^†‡^	19.4 ± 14.5^†‡§^	<0.001
Cereal-based dishes	15.0 ± 19.2	11.3 ± 17.8^†^	7.4 ± 15.8^†‡^	3.9 ± 11.5^†‡§^	<0.001
Breakfast cereals (ready to eat)	6.1 ± 10.7	6.1 ± 11.0	6.8 ± 11.2	8.9 ± 11.1^†‡§^	<0.001
Flours, cereals and starches	7.3 ± 16.4	6.7 ± 16.1	6.3 ± 15.6	3.1 ± 10.4^†‡§^	<0.001
Potatoes	5.2 ± 10.0	5.3 ± 10.0	5.5 ± 10.6	5.7 ± 9.5	0.036
Sweetened beverages	6.6 ± 11.2	4.5 ± 10.1^†^	2.8 ± 8.7^†‡^	1.4 ± 5.0^†‡^	<0.001
Sugar, honey and syrups	2.7 ± 5.1	4.2 ± 6.7^†^	4.3 ± 7.2^†^	5.2 ± 7.5^†^	<0.001
Cake-type dessert	3.0 ± 8.7	3.7 ± 9.8	4.3 ± 10.3	5.3 ± 11.4^†^	<0.001
Fruits and vegetables juices, cordials	3.9 ± 7.7	2.7 ± 6.6^†^	2.3 ± 5.8^†^	2.6 ± 6.3^†^	<0.001
Fancy breads	2.1 ± 6.3	2.8 ± 8.2	2.2 ± 7.5	1.9 ± 6.3	0.036
Sweet biscuits	1.9 ± 6.9	1.9 ± 4.4	2.4 ± 5.6	3.9 ± 6.5^†‡§^	<0.001
Dairy milk	1.8 ± 2.9	2.0 ± 2.7	2.4 ± 4.0^†^	3.0 ± 3.9^†‡^	<0.001
Tropical and subtropical fruit	1.6 ± 4.5	2.0 ± 5.1	2.3 ± 5.2	3.5 ± 6.2^†‡§^	<0.001
Pastries	2.2 ± 7.1	2.5 ± 7.4	2.7 ± 7.6	2.2 ± 6.0	0.760
Pome fruit	1.5 ± 3.5	1.9 ± 4.2	1.8 ± 3.8	2.2 ± 4.7	<0.001
Savoury biscuits	1.2 ± 5.4	1.8 ± 5.6	2.1 ± 6.4^†^	2.0 ± 5.1	<0.001
Poultry-based dishes	2.4 ± 7.2	1.8 ± 6.7	1.3 ± 5.4^†^	1.0 ± 4.5^†^	<0.001
Pastas	2.0 ± 7.6	1.5 ± 6.7	1.1 ± 5.4^†^	0.4 ± 2.8^†‡^	<0.001
Chocolates	1.4 ± 4.3	1.6 ± 5.0	1.8 ± 6.8	1.0 ± 3.2	0.283
Frozen milk products	1.0 ± 3.3	0.8 ± 3.2	1.2 ± 3.6	1.6 ± 4.6^‡^	<0.001
Other food groups	20.4 ± 18.1	21.2 ± 17.8	22.2 ± 17.9	22.1 ± 16.4	0.001

Values were presented as mean ± SD. Data were weighted to account for over- or under-sampling to enable representation of the general Australian population.

^1^*p* for trend across age groups tested by linear regression.

^†^Indicates *p* < 0.001 when compared with participants aged 19–30 years.

^‡^Indicates *p* < 0.001 when compared with participants aged 31–50 years.

^§^Indicates *p* < 0.001 when compared with participants aged 51–70 years.

**Table 4:** Per-consumer percentage dietary glycaemic load contribution from the top 20 food groups, stratified by age group.

Food groups	19–30 years	31–50 years	51–70 years	71 + years	*p*_trend_^1^
Bread and bread rolls	20.1 ± 11.7	21.3 ± 13.7	23.1 ± 14.9^†^	23.1 ± 12.8^†^	<0.001
Cereal-based dishes	29.6 ± 17.2	28.5 ± 17.7	29.8 ± 18.6	27.1 ± 17.0	0.859
Breakfast cereals (ready to eat)	18.8 ± 10.6	18.6 ± 11.5	18.0 ± 11.3	16.3 ± 10.2	<0.001
Flours, cereals and starches	34.5 ± 18.4	34.4 ± 19.4	33.8 ± 19.3	20.9 ± 18.9^†‡§^	<0.001
Potatoes	16.1 ± 11.8	16.2 ± 11.4	17.2 ± 12.1	15.1 ± 9.9	0.831
Sweetened beverages	16.1 ± 12.4	13.9 ± 13.5	12.4 ± 14.5^†^	11.0 ± 9.9	<0.001
Sugar, honey and syrups	6.8 ± 6.2	8.1 ± 7.4	8.8 ± 8.1^†^	9.6 ± 7.8^†^	<0.001
Cake-type dessert	18.8 ± 13.4	21.8 ± 13.1	21.7 ± 12.9	21.9 ± 13.5	0.015
Fruits and vegetables juices, cordials	12.5 ± 9.2	11.4 ± 9.3	9.9 ± 8.5^†^	9.9 ± 8.9	<0.001
Fancy breads	15.3 ± 9.2	19.4 ± 11.8	19.5 ± 12.2^†^	17.8 ± 9.6	0.108
Sweet biscuits	11.2 ± 13.5	8.4 ± 5.8	10.0 ± 7.4^†^	10.2 ± 6.7	0.921
Dairy milk	3.3 ± 3.3	2.8 ± 2.8	3.0 ± 4.3	3.5 ± 4.0	0.367
Tropical and subtropical fruit	9.2 ± 6.6	9.8 ± 7.3	9.8 ± 6.3	10.2 ± 6.5	0.192
Pastries	16.0 ± 12.1	15.3 ± 11.7	16.8 ± 11.1	14.1 ± 8.0	0.420
Pome fruit	7.0 ± 4.2	7.5 ± 5.2	7.4 ± 4.3	7.8 ± 5.7	0.026
Savoury biscuits	12.0 ± 12.8	11.1 ± 9.9	11.0 ± 11.0	9.3 ± 7.5	0.086
Poultry-based dishes	11.6 ± 12.2	12.1 ± 13.3	12.1 ± 11.5	13.3 ± 10.6	0.372
Pastas	22.0 ± 13.9	21.6 ± 14.2	22.0 ± 11.5	16.4 ± 9.2	0.489
Chocolates	8.0 ± 7.3	8.8 ± 8.5	9.9 ± 13.3	5.3 ± 5.7^§^	0.146
Frozen milk products	7.2 ± 5.9	7.1 ± 6.3	7.5 ± 5.7	7.5 ± 5.7	0.419
Other food groups	20.5 ± 18.1	21.2 ± 17.8	22.2 ± 17.9	22.1 ± 16.4	0.001

Values were presented as mean ± SD. Data were weighted to account for over- or under-sampling to enable representation of the general Australian population.

^1^*p* for trend across age groups tested by linear regression.

^†^Indicates *p* < 0.001 when compared with participants aged 19–30 years.

^‡^Indicates *p* < 0.001 when compared with participants aged 31–50 years.

^§^Indicates *p* < 0.001 when compared with participants aged 51–70 years.
