# Supplementary material for: Dietary glycaemic index and glycaemic load among Australian adults – results from the 2011–2012 Australian Health Survey
Source: Sci Rep. 2017 Mar 6;7:43882. doi: 10.1038/srep43882 (PMC5338341; doi:10.1038/srep43882)
Supplement: Supplementary Tables [file srep43882-s1.doc]

**Supplementary information**

**Title:** Dietary glycaemic index and glycaemic load among Australian adults – results from the 2011-2012 Australian Health Survey

**Authors:** Jimmy Chun Yu Louie, Molly Jones, Alan W Barclay, Jennie C Brand-Miller

**Supplementary Table 1** – List of abbreviated names of the top 20 glycaemic load contributing food groups

| **AUSNUT Food Group** | **Abbreviated Food Group** |
| --- | --- |
| Fruit and vegetables juices and drinks | Fruit and vegetables juices and drinks |
| Soft drinks, flavoured mineral waters and electrolyte drinks | Sweetened beverages |
| Flours and other cereal grains and starches | Flours, cereals and starches |
| Regular breads, and bread rolls (plain/unfilled/untopped varieties) | Bread and bread rolls |
| Breakfast cereals, ready to eat | Breakfast cereals (ready to eat) |
| Fancy breads, flat breads, English-style muffins and crumpets | Fancy breads |
| Pasta and pasta products | Pastas |
| Sweet biscuits | Sweet biscuits |
| Savoury biscuits | Savoury biscuits |
| Cakes, muffins, scones, cake-type desserts | Cake-type dessert |
| Pastries | Pastries |
| Mixed dishes where cereal is the major ingredient | Cereal-based dishes |
| Pome fruit | Pome fruit |
| Tropical and subtropical fruit | Tropical and subtropical fruit |
| Mixed dishes where poultry or feathered game is the major component | Poultry-based dishes |
| Dairy milk (cow, sheep and goat) | Dairy milk |
| Frozen milk products | Frozen milk products |
| Potatoes | Potatoes |
| Sugar, honey and syrups | Sugar, honey and syrups |
| Chocolate and chocolate-based confectionery | Chocolates |

**Supplementary Table 2 - Dietary intake of participants, stratified by age group (sensitivity analysis – all respondents included)**

|  | **19-30 years** | **31-50 years** | **51-70 years** | **71 years or above** | **^1^*p*_trend_** |
| --- | --- | --- | --- | --- | --- |
| Body mass index (BMI) - score measured | 25.3 ± 5.3 | 27.5 ± 5.4 | 28.6 ± 5.4 | 27.8 ± 5.0 | <0.001 |
| Dietary GI | 55.0 ± 7.0 | 54.1 ± 7.2 | 53.3 ± 7.6 | 53.5 ± 6.9 | <0.001 |
| Dietary GL | 141.6 ± 71.1 | 125.1 ± 63.3 | 111.7 ± 58.7 | 103.3 ± 47.0 | <0.001 |
| Dietary GL/MJ | 15.1 ± 4.4 | 14.3 ± 4.5 | 13.6 ± 4.5 | 14.2 ± 4.0 | <0.001 |
| Energy (kJ) | 9465.4 ± 4089.4 | 8871.9 ± 3738.5 | 8289.6 ± 3351.2 | 7295.2 ± 2753.7 | <0.001 |
| Energy from fat (%) | 31.2 ± 9.3 | 31.0 ± 8.7 | 30.7 ± 9.0 | 30.2 ± 8.8 | <0.001 |
| Energy from saturated fat (%) | 12.3 ± 4.9 | 12.1 ± 4.6 | 11.8 ± 4.9 | 12.3 ± 5.0 | 0.053 |
| Energy from protein (%) | 18.1 ± 6.5 | 18.4 ± 6.0 | 18.6 ± 6.4 | 18.4 ± 5.6 | 0.006 |
| Energy from carbohydrates (%) | 45.3 ± 10.7 | 43.4 ± 11.1 | 41.9 ± 11.4 | 44.0 ± 10.1 | <0.001 |
| Energy from total sugars (%) | 19.8 ± 9.5 | 19.3 ± 9.4 | 18.2 ± 8.8 | 20.1 ± 8.5 | 0.002 |
| Energy from starch (%) | 24.7 ± 9.9 | 23.3 ± 9.8 | 22.8 ± 9.8 | 23.1 ± 8.0 | <0.001 |
| Fibre density (g/MJ) | 2.5 ± 1.2 | 2.7 ± 1.3 | 3.0 ± 1.4 | 3.3 ± 1.9 | <0.001 |

Values were presented as mean ± SD. Data were weighted to account for over- or under-sampling to enable representation of the general Australian population.

^1^*P* values represent *P* for trend across age groups tested by linear regression for continuous variables, and for categorical variables the *p* values were tested by χ^2^ test.

**Supplementary Table 3 -** *Per-capita* percentage dietary glycaemic load contribution from the top 20 food groups, stratified by age group (sensitivity analysis – all respondents included)

| **Food groups** | **19-30 years** | **31-50 years** | **51-70 years** | **71 years or above** | **^1^*p*_trend_** |
| --- | --- | --- | --- | --- | --- |
| Bread and bread rolls | 12.4 ± 16.0 | 14.6 ± 16.8† | 18.2 ± 18.4†‡ | 20.7 ± 16.0†‡§ | <0.001 |
| Cereal-based dishes | 14.0 ± 19.2 | 10.9 ± 18.0† | 7.0 ± 15.7†‡ | 3.7 ± 11.4†‡§ | <0.001 |
| Breakfast cereals (ready to eat) | 6.0 ± 11.1 | 6.1 ± 11.4 | 7.1 ± 12.0 | 8.5 ± 11.4†‡ | <0.001 |
| Flours, cereals and starches | 7.4 ± 16.8 | 6.0 ± 15.4 | 5.8 ± 15.2 | 3.1 ± 10.5†‡§ | <0.001 |
| Potatoes | 4.9 ± 9.8 | 5.2 ± 10.8 | 5.3 ± 10.6 | 6.3 ± 11.3 | <0.001 |
| Sweetened beverages | 6.4 ± 11.4 | 4.4 ± 10.3† | 2.7 ± 8.7†‡ | 1.6 ± 5.7†‡ | <0.001 |
| Sugar, honey and syrups | 2.8 ± 6.0 | 4.4 ± 7.5† | 4.4 ± 7.5† | 5.1 ± 8.2† | <0.001 |
| Cake-type dessert | 2.4 ± 7.9 | 3.3 ± 9.4 | 3.7 ± 9.9† | 4.5 ± 10.8† | <0.001 |
| Fruits and vegetables juices, cordials | 4.0 ± 8.7 | 2.7 ± 7.0† | 2.3 ± 6.3† | 2.6 ± 6.4† | <0.001 |
| Fancy breads | 2.0 ± 6.3 | 2.8 ± 8.5† | 2.3 ± 7.8 | 1.8 ± 6.9‡ | 0.035 |
| Sweet biscuits | 1.7 ± 6.3 | 1.8 ± 4.5 | 2.5 ± 5.9†‡ | 3.9 ± 6.7†‡§ | <0.001 |
| Dairy milk | 1.9 ± 3.3 | 2.1 ± 3.4 | 2.3 ± 4.3† | 2.9 ± 3.8†‡§ | <0.001 |
| Tropical and subtropical fruit | 1.6 ± 4.9 | 2.2 ± 5.9 | 2.6 ± 6.4†§ | 3.5 ± 6.4†‡§ | <0.001 |
| Pastries | 2.1 ± 6.8 | 2.4 ± 7.4 | 2.5 ± 7.8 | 2.1 ± 6.2 | 0.531 |
| Pome fruit | 1.7 ± 4.4 | 2.1 ± 5.0 | 2.2 ± 4.8 | 2.3 ± 5.1† | <0.001 |
| Savoury biscuits | 1.3 ± 5.5 | 1.8 ± 5.9 | 2.2 ± 7.3† | 1.8 ± 4.9 | <0.001 |
| Poultry-based dishes | 2.4 ± 7.4 | 1.7 ± 6.4† | 1.3 ± 5.4† | 1.0 ± 4.7† | <0.001 |
| Pastas | 2.0 ± 8.1 | 1.3 ± 6.3† | 0.9 ± 5.0† | 0.7 ± 4.5† | <0.001 |
| Chocolates | 1.4 ± 4.4 | 1.5 ± 4.8 | 1.5 ± 5.9 | 0.9 ± 3.0 | 0.005 |
| Frozen milk products | 0.9 ± 3.1 | 0.9 ± 3.4 | 1.0 ± 3.5 | 1.4 ± 4.3†‡ | <0.001 |
| Other food groups | 20.6 ± 18.4 | 21.7 ± 18.7 | 22.3 ± 19.2 | 21.6 ± 17.2 | 0.005 |

Values were presented as mean ± SD. Data were weighted to account for over- or under-sampling to enable representation of the general Australian population.

^1^*P* for trend across age groups tested by linear regression

† indicates *p* < 0.001 when compared with participants aged 19-30 years

‡ indicates *p* < 0.001 when compared with participants aged 31-50 years

§ indicates *p* < 0.001 when compared with participants aged 51-70 years

**Supplementary Table 4 -** *Per-consumer* percentage dietary glycaemic load contribution from the top 20 food groups, stratified by age group (sensitivity analysis – all respondents included)

| **Food groups** | **19-30 years** | **31-50 years** | **51-70 years** | **71+ years** | **^1^*p*_trend_** |
| --- | --- | --- | --- | --- | --- |
| Bread and bread rolls | 22.9 ± 15.1 | 23.4 ± 15.7 | 25.9 ± 16.8†‡ | 24.9 ± 14.3 | <0.001 |
| Cereal-based dishes | 30.0 ± 17.6 | 29.3 ± 18.2 | 30.2 ± 18.8 | 28.0 ± 17.1 | 0.862 |
| Breakfast cereals (ready to eat) | 20.3 ± 11.3 | 19.4 ± 12.3 | 19.4 ± 12.5 | 17.0 ± 10.7† | <0.001 |
| Flours, cereals and starches | 35.9 ± 18.7 | 34.6 ± 19.7 | 34.1 ± 20.0 | 22.0 ± 19.4†‡§ | <0.001 |
| Potatoes | 16.1 ± 11.6 | 17.4 ± 13.2 | 17.8 ± 12.5 | 17.4 ± 12.5 | 0.007 |
| Sweetened beverages | 15.8 ± 13.3 | 14.0 ± 14.3 | 11.7 ± 15.0† | 11.9 ± 11.1 | <0.001 |
| Sugar, honey and syrups | 7.5 ± 7.9 | 8.7 ± 8.5 | 9.3 ± 8.5† | 10.3 ± 9.0† | <0.001 |
| Cake-type dessert | 18.8 ± 13.4 | 22.1 ± 13.5 | 21.6 ± 13.7 | 22.2 ± 13.6 | 0.008 |
| Fruits and vegetables juices, cordials | 13.5 ± 11.1 | 12.2 ± 10.3 | 10.7 ± 9.8† | 10.3 ± 9.2† | <0.001 |
| Fancy breads | 16.1 ± 9.9 | 20.7 ± 12.4† | 20.7 ± 12.8† | 19.9 ± 13.1 | 0.032 |
| Sweet biscuits | 10.8 ± 12.3 | 8.7 ± 6.2 | 11.0 ± 7.8‡ | 10.9 ± 7.0‡ | 0.010 |
| Dairy milk | 3.5 ± 3.8 | 3.0 ± 3.7 | 3.2 ± 4.7 | 3.4 ± 3.9 | 0.711 |
| Tropical and subtropical fruit | 10.0 ± 7.9 | 11.2 ± 6.7 | 11.5 ± 8.9 | 10.9 ± 6.9 | 0.489 |
| Pastries | 15.8 ± 11.5 | 16.0 ± 12.0 | 17.9 ± 12.6 | 15.3 ± 8.8 | 0.345 |
| Pome fruit | 8.3 ± 6.4 | 8.7 ± 6.9 | 8.9 ± 5.9 | 8.7 ± 6.5 | 0.077 |
| Savoury biscuits | 12.3 ± 12.2 | 11.8 ± 10.3 | 12.5 ± 13.1 | 9.2 ± 7.6 | 0.104 |
| Poultry-based dishes | 12.5 ± 12.6 | 12.2 ± 12.7 | 12.3 ± 12.0 | 13.3 ± 11.8 | 0.837 |
| Pastas | 22.8 ± 16.6 | 22.2 ± 14.2 | 20.5 ± 11.8 | 21.2 ± 14.7 | 0.365 |
| Chocolates | 8.4 ± 7.8 | 9.0 ± 8.6 | 9.8 ± 12.4 | 5.6 ± 5.8 | 0.036 |
| Frozen milk products | 7.2 ± 5.6 | 7.8 ± 6.8 | 7.7 ± 6.1 | 7.4 ± 7.1 | 0.440 |
| Other food groups | 20.6 ± 18.4 | 21.7 ± 18.7 | 22.3 ± 19.2 | 21.6 ± 17.2 | 0.005 |

Values were presented as mean ± SD. Data were weighted to account for over- or under-sampling to enable representation of the general Australian population.

^1^*P* for trend across age groups tested by linear regression

† indicates *p* < 0.001 when compared with participants aged 19-30 years

‡ indicates *p* < 0.001 when compared with participants aged 31-50 years

§ indicates *p* < 0.001 when compared with participants aged 51-70 years

**Supplementary Table 5** – Number of consumers of the top 20 glycaemic load contributing food groups, stratified by age and sex

| **Food Groups** | **19-30 years** | | **31-50 years** | | **51-70 years** | | **71+ years** | | **All adults** | |
| --- | --- | --- | --- | --- | --- | --- | --- | --- | --- | --- |
|  | **M** | **F** | **M** | **F** | **M** | **F** | **M** | **F** | **M** | **F** |
| Bread and bread rolls | 466 | 351 | 779 | 716 | 683 | 610 | 365 | 294 | 2193 | 1972 |
| Cereal-based dishes | 437 | 336 | 528 | 398 | 220 | 224 | 51 | 44 | 1236 | 1002 |
| Breakfast cereals (ready to eat) | 290 | 204 | 438 | 333 | 356 | 324 | 180 | 183 | 1264 | 1045 |
| Flours, cereals and starches | 203 | 118 | 224 | 230 | 182 | 154 | 42 | 55 | 651 | 558 |
| Potatoes | 286 | 203 | 398 | 374 | 336 | 239 | 127 | 123 | 1148 | 940 |
| Sweetened beverages | 390 | 237 | 451 | 316 | 250 | 160 | 41 | 41 | 1133 | 753 |
| Sugar, honey and syrups | 310 | 291 | 671 | 537 | 500 | 382 | 176 | 181 | 1658 | 1392 |
| Cake-type dessert | 115 | 125 | 179 | 221 | 174 | 179 | 76 | 84 | 544 | 608 |
| Fruits and vegetables juices and drinks | 283 | 188 | 308 | 243 | 223 | 187 | 81 | 94 | 895 | 712 |
| Fancy breads | 96 | 115 | 164 | 178 | 108 | 99 | 28 | 43 | 396 | 435 |
| Sweet biscuits | 137 | 118 | 277 | 244 | 220 | 212 | 112 | 146 | 747 | 721 |
| Dairy milk | 478 | 361 | 860 | 803 | 700 | 688 | 261 | 310 | 2299 | 2162 |
| Tropical and subtropical fruit | 143 | 129 | 249 | 224 | 185 | 241 | 91 | 137 | 668 | 731 |
| Pastries | 96 | 112 | 213 | 173 | 167 | 123 | 54 | 50 | 530 | 458 |
| Pome fruit | 166 | 162 | 301 | 291 | 226 | 212 | 86 | 104 | 779 | 768 |
| Savoury biscuits | 75 | 75 | 154 | 217 | 137 | 202 | 56 | 85 | 421 | 579 |
| Poultry-based dishes | 209 | 105 | 185 | 167 | 90 | 107 | 26 | 24 | 509 | 403 |
| Pastas | 90 | 51 | 89 | 77 | 42 | 47 | 7 | 8 | 228 | 182 |
| Chocolates | 111 | 157 | 211 | 224 | 161 | 161 | 62 | 59 | 546 | 601 |
| Frozen milk products | 118 | 85 | 164 | 115 | 169 | 125 | 77 | 66 | 527 | 391 |

**Supplementary Table 6 –** Ranking of the top 20 glycaemic load contributing food groups, stratified by age and sex

| **Food Groups** | **19-30 years** | | **31-50 years** | | **51-70 years** | | **71+**  **years** | | **All**  **adults** | |
| --- | --- | --- | --- | --- | --- | --- | --- | --- | --- | --- |
|  | **M** | **F** | **M** | **F** | **M** | **F** | **M** | **F** | **M** | **F** |
| Bread and bread rolls | 2 | 2 | 1 | 1 | 1 | 1 | 1 | 1 | 1 | 1 |
| Cereal-based dishes | 1 | 1 | 2 | 2 | 4 | 2 | 6 | 9 | 2 | 2 |
| Breakfast cereals (ready to eat) | 5 | 6 | 4 | 4 | 2 | 3 | 2 | 2 | 3 | 3 |
| Flours, cereals and starches | 3 | 5 | 3 | 3 | 3 | 4 | 8 | 10 | 4 | 4 |
| Potatoes | 6 | 4 | 6 | 5 | 5 | 6 | 3 | 4 | 5 | 5 |
| Sweetened beverages | 4 | 3 | 5 | 8 | 8 | 16 | 16 | 17 | 6 | 8 |
| Sugar, honey and syrups | 9 | 9 | 7 | 7 | 6 | 7 | 4 | 5 | 7 | 7 |
| Cake-type dessert | 12 | 7 | 8 | 6 | 7 | 5 | 5 | 3 | 8 | 6 |
| Fruits and vegetables juices and drinks | 7 | 8 | 9 | 10 | 11 | 14 | 12 | 11 | 9 | 9 |
| Fancy breads | 15 | 11 | 11 | 9 | 13 | 12 | 14 | 15 | 12 | 10 |
| Sweet biscuits | 11 | 17 | 14 | 16 | 10 | 11 | 7 | 7 | 11 | 14 |
| Dairy milk | 13 | 15 | 12 | 15 | 12 | 10 | 10 | 8 | 13 | 13 |
| Tropical and subtropical fruit | 16 | 14 | 15 | 13 | 14 | 8 | 9 | 6 | 14 | 11 |
| Pastries | 14 | 10 | 10 | 11 | 9 | 13 | 11 | 14 | 10 | 12 |
| Pome fruit | 17 | 16 | 16 | 14 | 17 | 15 | 13 | 12 | 16 | 16 |
| Savoury biscuits | 18 | 19 | 19 | 12 | 15 | 9 | 17 | 13 | 18 | 15 |
| Poultry-based dishes | 8 | 13 | 13 | 18 | 20 | 18 | 18 | 18 | 15 | 18 |
| Pastas | 10 | 18 | 17 | 19 | 19 | 19 | 20 | 20 | 17 | 19 |
| Chocolates | 20 | 12 | 18 | 17 | 16 | 17 | 19 | 19 | 19 | 17 |
| Frozen milk products | 19 | 20 | 20 | 20 | 18 | 20 | 15 | 16 | 20 | 20 |

**Supplementary Table 7 –** per capita contribution to dietary glycaemic load by the top 20 food groups, stratified by sex and age group

|  | **19-30 years** | | ***p* value*** | **31-50 years** | | ***p* value*** | **51-70 years** | | ***p* value*** | **71+ years** | | ***p* value*** | **All adults** | | ***p* value*** |
| --- | --- | --- | --- | --- | --- | --- | --- | --- | --- | --- | --- | --- | --- | --- | --- |
| **Food groups** | **Men** | **Women** |  | **Men** | **Women** |  | **Men** | **Women** |  | **Men** | **Women** |  | **Men** | **Women** |  |
| Bread and bread rolls | 10.8 ± 12.7 | 10.8 ± 13.8 | 0.994 | 13.9 ± 15.6 | 13.3 ± 14.3 | 0.277 | 17.6 ± 16.5 | 15.7 ± 16.2 | 0.015 | 20.6 ± 14.7 | 18.4 ± 14.2 | 0.050 | 14.8 ± 15.4 | 14.0 ± 14.9 | 0.053 |
| Cereal-based dishes | 14.9 ± 19.1 | 15.1 ± 19.3 | 0.826 | 12.0 ± 18.0 | 10.4 ± 17.5 | 0.030 | 7.1 ± 16.0 | 7.7 ± 15.7 | 0.446 | 4.4 ± 12.5 | 3.4 ± 10.5 | 0.227 | 10.7 ± 17.7 | 9.8 ± 17.1 | 0.052 |
| Breakfast cereals (ready to eat) | 6.8 ± 11.5 | 5.2 ± 9.5 | 0.004 | 6.5 ± 11.2 | 5.7 ± 10.8 | 0.067 | 7.3 ± 11.7 | 6.4 ± 10.6 | 0.101 | 9.9 ± 11.4 | 8.0 ± 10.7 | 0.032 | 7.1 ± 11.4 | 6.1 ± 10.5 | 0.000 |
| Flours, cereals and starches | 8.8 ± 18.1 | 5.3 ± 13.7 | 0.000 | 6.6 ± 16.2 | 6.8 ± 15.9 | 0.742 | 7.2 ± 16.8 | 5.4 ± 14.2 | 0.016 | 2.8 ± 9.9 | 3.3 ± 10.8 | 0.557 | 7.0 ± 16.5 | 5.6 ± 14.4 | 0.001 |
| Potatoes | 5.0 ± 9.6 | 5.4 ± 10.6 | 0.468 | 5.4 ± 10.0 | 5.3 ± 10.1 | 0.748 | 6.6 ± 11.9 | 4.4 ± 8.9 | 0.000 | 6.0 ± 9.5 | 5.4 ± 9.5 | 0.390 | 5.7 ± 10.4 | 5.1 ± 9.8 | 0.017 |
| Sweetened beverages | 7.3 ± 10.9 | 5.7 ± 11.4 | 0.007 | 5.6 ± 10.7 | 3.4 ± 9.2 | 0.000 | 3.7 ± 10.0 | 2.0 ± 6.8 | 0.000 | 1.5 ± 5.1 | 1.2 ± 5.0 | 0.478 | 5.1 ± 10.3 | 3.2 ± 8.9 | 0.000 |
| Fruit and vegetable juices and drinks | 4.1 ± 8.0 | 3.6 ± 7.4 | 0.072 | 2.8 ± 6.6 | 2.6 ± 6.6 | 0.366 | 2.4 ± 6.0 | 2.1 ± 5.6 | 0.000 | 2.1 ± 4.9 | 3.0 ± 7.3 | 0.252 | 2.9 ± 6.7 | 2.7 ± 6.6 | 0.047 |
| Cake-type desserts | 2.0 ± 5.9 | 4.3 ± 11.1 | 0.000 | 2.9 ± 8.5 | 4.6 ± 11.0 | 0.000 | 4.0 ± 9.7 | 4.6 ± 11.0 | 0.197 | 4.7 ± 9.8 | 5.8 ± 12.7 | 0.212 | 3.1 ± 8.5 | 4.7 ± 11.2 | 0.000 |
| Sweet biscuits | 2.0 ± 8.3 | 1.6 ± 4.5 | 0.193 | 1.8 ± 4.2 | 1.9 ± 4.6 | 0.548 | 2.5 ± 5.8 | 2.4 ± 5.5 | 0.281 | 3.9 ± 6.6 | 4.0 ± 6.3 | 0.058 | 2.2 ± 6.2 | 2.2 ± 5.1 | 0.195 |
| Savoury biscuits | 1.1 ± 5.5 | 1.2 ± 5.2 | 0.000 | 1.3 ± 4.9 | 2.2 ± 6.4 | 0.039 | 1.6 ± 5.6 | 2.5 ± 7.2 | 0.725 | 1.5 ± 4.1 | 2.4 ± 5.9 | 0.561 | 1.4 ± 5.2 | 2.1 ± 6.4 | 0.001 |
| Fancy breads | 1.6 ± 5.5 | 2.7 ± 7.0 | 0.271 | 2.5 ± 7.8 | 3.2 ± 8.7 | 0.384 | 2.2 ± 7.2 | 2.3 ± 7.7 | 0.632 | 1.7 ± 6.3 | 2.0 ± 6.3 | 0.876 | 2.1 ± 7.0 | 2.7 ± 7.8 | 0.986 |
| Dairy milk | 1.9 ± 2.6 | 1.7 ± 3.2 | 0.290 | 1.9 ± 2.6 | 2.0 ± 2.7 | 0.331 | 2.2 ± 4.0 | 2.5 ± 4.0 | 0.203 | 2.4 ± 3.0 | 3.4 ± 4.5 | 0.001 | 2.0 ± 3.1 | 2.3 ± 3.5 | 0.012 |
| Pome fruit | 1.4 ± 3.4 | 1.7 ± 3.5 | 0.166 | 1.7 ± 4.1 | 2.1 ± 4.3 | 0.041 | 1.6 ± 3.5 | 2.0 ± 4.2 | 0.000 | 2.0 ± 4.4 | 2.4 ± 4.9 | 0.001 | 1.6 ± 3.8 | 2.0 ± 4.2 | 0.000 |
| Tropical and subtropical fruit | 1.5 ± 4.1 | 1.8 ± 5.0 | 0.002 | 1.8 ± 4.6 | 2.2 ± 5.7 | 0.343 | 1.8 ± 4.4 | 2.8 ± 5.8 | 0.007 | 2.7 ± 5.3 | 4.2 ± 6.8 | 0.466 | 1.8 ± 4.5 | 2.5 ± 5.8 | 0.448 |
| Pastas | 2.4 ± 8.3 | 1.6 ± 6.7 | 0.093 | 1.5 ± 6.1 | 1.6 ± 7.3 | 0.037 | 1.1 ± 5.7 | 1.0 ± 5.1 | 0.056 | 0.3 ± 2.3 | 0.4 ± 3.1 | 0.325 | 1.5 ± 6.4 | 1.3 ± 6.2 | 0.000 |
| Pastries | 1.7 ± 6.4 | 2.8 ± 7.8 | 0.774 | 2.6 ± 7.5 | 2.4 ± 7.3 | 0.000 | 3.2 ± 8.3 | 2.2 ± 6.8 | 0.003 | 2.4 ± 6.2 | 2.0 ± 5.8 | 0.031 | 2.5 ± 7.4 | 2.4 ± 7.1 | 0.000 |
| Sugar, honey and syrups | 2.5 ± 5.2 | 3.0 ± 5.0 | 0.009 | 4.3 ± 6.9 | 4.1 ± 6.5 | 0.882 | 4.9 ± 7.3 | 3.7 ± 7.0 | 0.024 | 5.5 ± 7.5 | 4.9 ± 7.4 | 0.476 | 4.1 ± 6.8 | 3.8 ± 6.5 | 0.644 |
| Poultry-based dishes | 2.8 ± 7.9 | 1.8 ± 6.3 | 0.054 | 1.8 ± 6.8 | 1.8 ± 6.7 | 0.635 | 1.0 ± 4.4 | 1.6 ± 6.2 | 0.752 | 0.9 ± 3.5 | 1.1 ± 5.3 | 0.697 | 1.8 ± 6.3 | 1.7 ± 6.3 | 0.214 |
| Chocolates | 0.8 ± 2.8 | 2.2 ± 5.5 | 0.000 | 1.4 ± 4.4 | 1.9 ± 5.6 | 0.016 | 1.6 ± 6.5 | 1.9 ± 7.1 | 0.354 | 0.9 ± 2.5 | 1.1 ± 3.6 | 0.384 | 1.3 ± 4.7 | 1.9 ± 5.9 | 0.000 |
| Frozen milk products | 1.0 ± 3.0 | 1.0 ± 3.6 | 0.939 | 0.9 ± 3.2 | 0.8 ± 3.2 | 0.354 | 1.4 ± 4.0 | 1.0 ± 3.1 | 0.008 | 1.6 ± 3.9 | 1.6 ± 5.2 | 0.997 | 1.1 ± 3.5 | 1.0 ± 3.6 | 0.073 |
| Other food groups | 19.7 ± 17.6 | 21.4 ± 18.7 | 0.065 | 20.7 ± 17.6 | 21.8 ± 18.0 | 0.161 | 18.8 ± 15.9 | 25.7 ± 19.2 | 0.000 | 22.2 ± 16.7 | 22.1 ± 16.1 | 0.937 | 20.1 ± 17.1 | 22.9 ± 18.4 | 0.000 |

**p* value for difference between men and women in the same age group, tested by one-way ANOVA.

**Supplementary Table 8 – per consumer contribution to dietary glycaemic load by the top 20 food groups, stratified by sex and age group**

|  | **19-30 years** | | ***p* value*** | **31-50 years** | | ***p* value*** | **51-70 years** | | ***p* value*** | **71+ years** | | ***p* value*** | **All adults** | | ***p* value*** |
| --- | --- | --- | --- | --- | --- | --- | --- | --- | --- | --- | --- | --- | --- | --- | --- |
| **Food groups** | **Men** | **Women** |  | **Men** | **Women** |  | **Men** | **Women** |  | **Men** | **Women** |  | **Men** | **Women** |  |
| Bread and bread rolls | 19.7 ± 10.9 | 20.6 ± 12.7 | 0.273 | 22.1 ± 14.4 | 20.6 ± 12.9 | 0.033 | 23.7 ± 14.9 | 22.4 ± 14.9 | 0.141 | 24.0 ± 13.1 | 22.3 ± 12.6 | 0.114 | 22.3 ± 13.8 | 21.4 ± 13.5 | 0.035 |
| Cereal-based dishes | 29.1 ± 17.4 | 30.2 ± 16.9 | 0.373 | 28.1 ± 17.5 | 29.1 ± 17.8 | 0.427 | 29.7 ± 19.9 | 29.9 ± 17.2 | 0.916 | 27.1 ± 18.3 | 27.2 ± 15.7 | 0.984 | 28.7 ± 18.0 | 29.6 ± 17.3 | 0.263 |
| Breakfast cereals (ready to eat) | 19.9 ± 11.2 | 17.2 ± 9.5 | 0.004 | 18.4 ± 11.5 | 19.0 ± 11.6 | 0.488 | 18.8 ± 11.7 | 17.2 ± 10.8 | 0.071 | 16.9 ± 10.2 | 15.6 ± 10.2 | 0.221 | 18.6 ± 11.3 | 17.5 ± 10.8 | 0.012 |
| Flours, cereals and starches | 37.0 ± 18.2 | 30.2 ± 17.8 | 0.001 | 36.2 ± 19.5 | 32.7 ± 19.2 | 0.051 | 36.3 ± 19.1 | 30.7 ± 19.3 | 0.008 | 20.6 ± 18.7 | 21.2 ± 19.3 | 0.879 | 35.5 ± 19.3 | 30.5 ± 19.1 | 0.000 |
| Potatoes | 14.9 ± 11.2 | 17.8 ± 12.4 | 0.007 | 16.8 ± 10.8 | 15.6 ± 11.9 | 0.152 | 17.9 ± 13.4 | 16.2 ± 9.8 | 0.095 | 14.6 ± 9.7 | 15.6 ± 10.0 | 0.451 | 16.4 ± 11.7 | 16.2 ± 11.3 | 0.726 |
| Sweetened beverages | 16.1 ± 11.3 | 16.1 ± 14.0 | 0.970 | 15.3 ± 12.9 | 11.9 ± 14.1 | 0.000 | 13.5 ± 15.5 | 10.8 ± 12.7 | 0.061 | 11.3 ± 9.2 | 10.7 ± 10.7 | 0.798 | 15.0 ± 12.9 | 12.9 ± 13.8 | 0.001 |
| Fruit and vegetable juices and drinks | 12.4 ± 9.4 | 12.7 ± 8.7 | 0.710 | 11.1 ± 9.1 | 11.9 ± 9.4 | 0.316 | 9.9 ± 8.6 | 9.8 ± 8.4 | 0.943 | 8.0 ± 6.5 | 11.5 ± 10.2 | 0.010 | 10.9 ± 9.0 | 11.5 ± 9.1 | 0.201 |
| Cake-type desserts | 14.4 ± 9.0 | 22.9 ± 15.5 | 0.000 | 20.3 ± 12.2 | 23.0 ± 13.8 | 0.045 | 20.9 ± 12.0 | 22.4 ± 13.7 | 0.263 | 18.9 ± 11.1 | 24.6 ± 14.9 | 0.008 | 19.0 ± 11.6 | 23.0 ± 14.3 | 0.000 |
| Sweet biscuits | 12.8 ± 17.1 | 9.3 ± 6.7 | 0.035 | 8.0 ± 5.6 | 8.8 ± 6.0 | 0.082 | 10.4 ± 7.5 | 9.7 ± 7.3 | 0.335 | 10.8 ± 6.9 | 9.7 ± 6.5 | 0.207 | 10.0 ± 9.6 | 9.3 ± 6.6 | 0.132 |
| Savoury biscuits | 13.1 ± 13.8 | 10.9 ± 11.8 | 0.300 | 10.6 ± 9.6 | 11.5 ± 10.1 | 0.380 | 11.0 ± 10.3 | 11.0 ± 11.5 | 0.962 | 8.3 ± 5.9 | 10.0 ± 8.4 | 0.205 | 10.9 ± 10.4 | 11.0 ± 10.6 | 0.829 |
| Fancy breads | 14.6 ± 9.7 | 15.9 ± 8.8 | 0.310 | 18.8 ± 12.1 | 19.9 ± 11.6 | 0.388 | 18.6 ± 11.8 | 20.5 ± 12.5 | 0.269 | 19.2 ± 10.6 | 16.8 ± 8.9 | 0.317 | 17.8 ± 11.5 | 18.7 ± 11.0 | 0.242 |
| Dairy milk | 3.4 ± 2.7 | 3.2 ± 3.9 | 0.367 | 2.7 ± 2.7 | 2.8 ± 2.8 | 0.722 | 2.9 ± 4.3 | 3.1 ± 4.3 | 0.389 | 2.9 ± 3.0 | 3.9 ± 4.6 | 0.002 | 3.0 ± 3.3 | 3.1 ± 3.8 | 0.099 |
| Pome fruit | 7.0 ± 4.5 | 6.9 ± 3.9 | 0.879 | 7.1 ± 5.5 | 8.0 ± 4.9 | 0.039 | 6.7 ± 3.8 | 8.2 ± 4.6 | 0.000 | 7.3 ± 5.6 | 8.2 ± 5.7 | 0.280 | 7.0 ± 4.9 | 7.8 ± 4.8 | 0.000 |
| Tropical and subtropical fruit | 8.9 ± 5.8 | 9.5 ± 7.4 | 0.462 | 8.8 ± 6.4 | 11.0 ± 8.0 | 0.001 | 9.2 ± 5.5 | 10.3 ± 6.9 | 0.088 | 9.1 ± 6.1 | 11.0 ± 6.7 | 0.035 | 9.0 ± 6.0 | 10.5 ± 7.3 | 0.000 |
| Pastas | 22.3 ± 14.1 | 21.3 ± 13.6 | 0.679 | 20.4 ± 11.8 | 22.9 ± 16.5 | 0.260 | 24.9 ± 11.3 | 19.5 ± 11.2 | 0.029 | 13.8 ± 7.4 | 18.9 ± 10.5 | 0.302 | 21.8 ± 12.7 | 21.4 ± 14.2 | 0.795 |
| Pastries | 14.8 ± 13.1 | 17.0 ± 11.2 | 0.207 | 15.5 ± 11.4 | 15.1 ± 12.2 | 0.749 | 17.6 ± 11.4 | 15.8 ± 10.7 | 0.181 | 13.6 ± 8.2 | 14.6 ± 7.8 | 0.552 | 15.8 ± 11.5 | 15.7 ± 11.1 | 0.832 |
| Sugar, honey and syrups | 6.8 ± 6.7 | 6.8 ± 5.6 | 0.984 | 7.9 ± 7.7 | 8.4 ± 7.1 | 0.279 | 9.1 ± 7.8 | 8.4 ± 8.5 | 0.201 | 9.7 ± 7.7 | 9.6 ± 8.0 | 0.900 | 8.2 ± 7.6 | 8.2 ± 7.4 | 0.885 |
| Poultry-based dishes | 11.5 ± 12.5 | 11.7 ± 11.7 | 0.889 | 12.0 ± 13.6 | 12.2 ± 13.1 | 0.863 | 10.8 ± 9.8 | 13.2 ± 12.7 | 0.145 | 10.4 ± 7.1 | 16.4 ± 12.8 | 0.045 | 11.5 ± 12.2 | 12.6 ± 12.6 | 0.183 |
| Chocolates | 6.2 ± 5.7 | 9.3 ± 8.1 | 0.001 | 8.2 ± 7.6 | 9.4 ± 9.3 | 0.131 | 9.4 ± 13.0 | 10.5 ± 13.6 | 0.446 | 4.3 ± 4.2 | 6.5 ± 6.7 | 0.028 | 7.7 ± 9.1 | 9.4 ± 10.2 | 0.003 |
| Frozen milk products | 7.0 ± 4.8 | 7.6 ± 7.2 | 0.489 | 6.8 ± 6.0 | 7.6 ± 6.8 | 0.356 | 7.9 ± 6.1 | 6.9 ± 5.2 | 0.143 | 6.3 ± 5.6 | 8.5 ± 9.4 | 0.090 | 7.1 ± 5.7 | 7.5 ± 7.0 | 0.377 |
| Other food groups | 19.7 ± 17.6 | 21.4 ± 18.7 | 0.065 | 20.7 ± 17.6 | 21.8 ± 18.0 | 0.161 | 18.8 ± 15.9 | 25.7 ± 19.1 | 0.000 | 22.2 ± 16.7 | 22.1 ± 16.1 | 0.937 | 20.1 ± 17.1 | 22.9 ± 18.4 | 0.000 |

**p* value for difference between men and women in the same age group, tested by one-way ANOVA.

**Supplementary Table 9 -** Per-capita percentage dietary glycaemic load contribution from the top 20 food groups, stratified by age group and adjusted for energy and SEIFA

| **Food groups** | **19-30 years** | **31-50 years** | **51-70 years** | **71 years or above** | **^1^*p*_trend_** |
| --- | --- | --- | --- | --- | --- |
| Bread and bread rolls | 11.1 ± 0.5 | 13.8 ± 0.4 | 16.5 ± 0.5 | 18.6 ± 0.6 | <0.001 |
| Cereal-based dishes | 14.9 ± 0.8 | 11.2 ± 0.5 | 7.5 ± 0.5 | 4.3 ± 0.5 | <0.001 |
| Breakfast cereals (ready to eat) | 6.1 ± 0.5 | 6.1 ± 0.3 | 6.9 ± 0.3 | 8.9 ± 0.5 | <0.001 |
| Flours, cereals and starches | 7.1 ± 0.7 | 6.7 ± 0.5 | 6.4 ± 0.6 | 3.4 ± 0.5 | 0.001 |
| Potatoes | 5.1 ± 0.4 | 5.4 ± 0.3 | 5.5 ± 0.3 | 5.7 ± 0.4 | 0.092 |
| Sweetened beverages | 6.5 ± 0.5 | 4.6 ± 0.3 | 2.9 ± 0.2 | 1.5 ± 0.2 | <0.001 |
| Sugar, honey and syrups | 2.7 ± 0.2 | 4.3 ± 0.2 | 4.3 ± 0.2 | 5.0 ± 0.3 | <0.001 |
| Cake-type dessert | 2.7 ± 0.3 | 3.6 ± 0.2 | 4.4 ± 0.3 | 5.8 ± 0.5 | <0.001 |
| Fruits and vegetables juices, cordials | 3.9 ± 0.3 | 2.7 ± 0.2 | 2.2 ± 0.2 | 2.5 ± 0.3 | <0.001 |
| Fancy breads | 2.2 ± 0.2 | 2.8 ± 0.2 | 2.2 ± 0.2 | 1.8 ± 0.3 | 0.036 |
| Sweet biscuits | 1.9 ± 0.4 | 1.9 ± 0.1 | 2.4 ± 0.2 | 3.9 ± 0.3 | 0.003 |
| Dairy milk | 1.9 ± 0.1 | 2.0 ± 0.1 | 2.3 ± 0.1 | 2.8 ± 0.2 | <0.001 |
| Tropical and subtropical fruit | 1.7 ± 0.2 | 2.0 ± 0.1 | 2.3 ± 0.1 | 3.3 ± 0.3 | <0.001 |
| Pastries | 2.1 ± 0.3 | 2.5 ± 0.2 | 2.7 ± 0.2 | 2.2 ± 0.3 | 0.681 |
| Pome fruit | 1.6 ± 0.1 | 1.9 ± 0.1 | 1.8 ± 0.1 | 2.1 ± 0.2 | 0.073 |
| Savoury biscuits | 1.2 ± 0.2 | 1.8 ± 0.2 | 2.1 ± 0.2 | 1.8 ± 0.2 | 0.001 |
| Poultry-based dishes | 2.4 ± 0.3 | 1.8 ± 0.2 | 1.3 ± 0.2 | 1.0 ± 0.2 | <0.001 |
| Pastas | 2.0 ± 0.3 | 1.5 ± 0.2 | 1.1 ± 0.2 | 0.3 ± 0.1 | <0.001 |
| Chocolates | 1.4 ± 0.2 | 1.6 ± 0.1 | 1.8 ± 0.2 | 1.0 ± 0.1 | 0.391 |
| Frozen milk products | 0.9 ± 0.1 | 0.8 ± 0.1 | 1.2 ± 0.1 | 1.7 ± 0.2 | <0.001 |
| Other food groups | 20.4 ± 0.7 | 21.1 ± 0.5 | 22.2 ± 0.5 | 22.2 ± 0.7 | 0.008 |

Values were presented as mean ± SEM. Data were weighted to account for over- or under-sampling to enable representation of the general Australian population.

^1^*P* for trend across age groups tested by linear regression

**Supplementary Table 10 -** Per-consumer percentage dietary glycaemic load contribution from the top 20 food groups, stratified by age group and adjusted for energy and SEIFA

| **Food groups** | **19-30 years** | **31-50 years** | **51-70 years** | **71+ years** | **^1^*p*_trend_** |
| --- | --- | --- | --- | --- | --- |
| Bread and bread rolls | 20.9 ± 0.6 | 21.7 ± 0.5 | 22.8 ± 0.5 | 21.5 ± 0.6 | 0.179 |
| Cereal-based dishes | 29.9 ± 1.0 | 28.4 ± 0.7 | 29.6 ± 1.2 | 26.5 ± 1.8 | 0.535 |
| Breakfast cereals (ready to eat) | 19.2 ± 0.8 | 18.8 ± 0.5 | 17.9 ± 0.5 | 15.7 ± 0.7 | <0.001 |
| Flours, cereals and starches | 34.2 ± 1.6 | 34.6 ± 1.2 | 33.7 ± 1.6 | 21.1 ± 2.1 | 0.001 |
| Potatoes | 16.4 ± 0.9 | 16.2 ± 0.5 | 17.1 ± 0.6 | 14.6 ± 0.7 | 0.616 |
| Sweetened beverages | 16.1 ± 0.8 | 13.9 ± 0.6 | 12.4 ± 0.9 | 11.1 ± 1.1 | <0.001 |
| Sugar, honey and syrups | 6.9 ± 0.4 | 8.2 ± 0.3 | 8.7 ± 0.3 | 9.2 ± 0.5 | <0.001 |
| Cake-type dessert | 19.2 ± 1.4 | 21.7 ± 0.8 | 21.6 ± 0.8 | 21.6 ± 1.2 | 0.150 |
| Fruits and vegetables juices, cordials | 12.8 ± 0.7 | 11.5 ± 0.5 | 9.8 ± 0.5 | 9.1 ± 0.9 | <0.001 |
| Fancy breads | 15.9 ± 0.9 | 19.3 ± 0.9 | 19.5 ± 1.1 | 16.6 ± 1.3 | 0.580 |
| Sweet biscuits | 11.6 ± 2.0 | 8.6 ± 0.3 | 10.0 ± 0.4 | 9.5 ± 0.5 | 0.736 |
| Dairy milk | 3.4 ± 0.2 | 2.8 ± 0.1 | 3.0 ± 0.1 | 3.3 ± 0.2 | 0.576 |
| Tropical and subtropical fruit | 9.7 ± 0.6 | 10.1 ± 0.4 | 9.7 ± 0.3 | 9.4 ± 0.5 | 0.402 |
| Pastries | 16.2 ± 1.2 | 15.5 ± 0.7 | 16.7 ± 0.8 | 13.3 ± 0.8 | 0.195 |
| Pome fruit | 7.1 ± 0.3 | 7.7 ± 0.3 | 7.4 ± 0.2 | 7.0 ± 0.4 | 0.761 |
| Savoury biscuits | 12.1 ± 1.4 | 11.4 ± 0.7 | 10.9 ± 0.8 | 8.7 ± 0.7 | 0.044 |
| Poultry-based dishes | 11.9 ± 1.1 | 12.1 ± 0.9 | 11.9 ± 1.1 | 12.2 ± 1.4 | 0.985 |
| Pastas | 22.4 ± 2.1 | 21.6 ± 1.8 | 21.4 ± 1.6 | 16.2 ± 2.1 | 0.429 |
| Chocolates | 8.2 ± 0.7 | 8.9 ± 0.5 | 9.9 ± 0.9 | 4.9 ± 0.5 | 0.072 |
| Frozen milk products | 7.4 ± 0.5 | 7.2 ± 0.4 | 7.4 ± 0.5 | 7.0 ± 0.7 | 0.993 |
| Other food groups | 20.5 ± 0.7 | 21.2 ± 0.5 | 22.2 ± 0.5 | 22.2 ± 0.7 | 0.010 |

Values were presented as mean ± SEM. Data were weighted to account for over- or under-sampling to enable representation of the general Australian population.

^1^*P* for trend across age groups tested by linear regression
